# Characterizing the Immature Immunophenotype of Sickle Cell Disease Monocytes

**DOI:** 10.7759/cureus.60703

**Published:** 2024-05-20

**Authors:** Luke Gingell, Borys Hrinczenko

**Affiliations:** 1 Medical School, Michigan State University, Grand Rapids, USA; 2 Hematology/Oncology, Michigan State University, East Lansing, USA

**Keywords:** immunology and research, inflammatory cytokines, vascular endothelium, sickle cell disease: scd, monocytes

## Abstract

Sickle cell disease (SCD) is marked by episodic vaso-occlusive crisis (VOC). Recurrent VOC creates a pro-inflammatory state that induces phenotypic alterations in innate immune cells. Monocytes are of particular interest to VOC pathophysiology because they are especially malleable to inflammatory signaling. Indeed, inflammatory disease states such as chronic obstructive pulmonary disease (COPD), obesity and atherosclerosis are known to influence monocyte development and alter monocyte subpopulations. In this study, we describe SCD monocyte subsets by performing immunophenotypic flow cytometric, enzymatic, and morphologic analysis on peripheral blood. Herein, we add to the growing body of evidence suggesting aberrant monocyte populations underpin VOC pathophysiology. We found that SCD monocytes possess an immature phenotype as demonstrated by 1) decreased CD4 positivity (p < .01), 2) low α-naphthyl butyrate esterase (ANBE) expression, and 3) naïve morphologic features. We additionally found an increase in CD14^+^CD16^-^CD4^-^ monocytes (p < .01), a subset associated with the impaired immune response of post-trauma patients. Interestingly, we also found a large proportion of CD14^+^CD4^-^HLA-DR^-^ monocytes which, under normal circumstances, are exclusively found in neonates (p < .01). Finally, we report an increase in nonclassical monocytes (CD14^dim^CD16^+^), a subset recently shown to have a critical role in prevention and recovery from VOC.

## Introduction

The recurrent vaso-occlusive crisis (VOC) is the hallmark of sickle cell disease (SCD). Painful VOC is the leading cause of acute care utilization and hospitalization in SCD patients, heavily contributing to the morbidity and mortality of this disease [1,2]. SCD has long been known to originate from a single nucleotide mutation of the b-globin gene, leading to polymerization of the abnormal hemoglobin S (HbS), which results in vascular obstruction by sickle red blood cells (RBC). However, SCD pathophysiology is now understood to be more complex, involving phenotypic alterations in members of both the innate and adaptive immune systems.

The majority of immune derangement observed in SCD is thought to be due to dysfunction of the spleen [3]. When sickling occurs in the spleen, this organ undergoes episodic auto-infarction that begins in early infancy and leads to a rapid loss of splenic function (hyposplenism) [3,4]. Without a functioning spleen, SCD patients have reduced opsonophagocytic function and thus are unable to clear bacteria from the blood, increasing susceptibility to severe, recurrent bacterial infections [4,5]. However, the role of immune cells in SCD pathophysiology cannot be entirely explained by hyposplenism. 

It has been widely reported that recurrent sickling creates a pro-inflammatory state, causing SCD to have an immune profile similar to a chronic inflammatory condition [6,7]. Importantly, innate immune cells (monocytes, neutrophils, basophils, eosinophils, natural killer (NK) cells, platelets, macrophages, and mast cells) have been implicated as drivers of inflammation in SCD [6,8,9]. Previous work suggests that neutrophils play a central role in vaso-occlusion through their interactions with erythrocytes and vascular endothelium [10,11]. SCD patients' neutrophils display an activated phenotype with increased adhesive properties that amplify during a VOC [12]. Thus, SCD neutrophils are thought to actively contribute to the genesis of VOC. This hypothesis is substantiated by the clinical correlation between absolute neutrophil count and SCD severity [13]. While the function of neutrophils in SCD pathophysiology is established, the role of monocytes in SCD remains incompletely understood.

Monocytes are a heterogeneous population of innate immune cells that make up one component of the mononuclear phagocyte system (MPS), which includes macrophages and dendritic cells [14]. Three major monocyte subpopulations have been identified: classical (CD14+CD16-), intermediate (CD14+CD16+), and non-classical (CD14dimCD16+) [15-17]. Monocytes are thought to develop in a linear trajectory from classical to intermediate to non-classical [18-20]. These subsets are functionally distinguished from one another by their responses to homeostatic and pathologic stimuli [20,21]. This cellular plasticity is partly achieved through distinct methylation patterns between subsets that results in varied surface receptor expression, affording each subpopulation a unique set of characteristics [22]. However, severe inflammation can modulate monocytic developmental pathways, increasing the number of monocytes and altering their functional specialization [23,24]. Indeed, phenotypic changes in monocyte subpopulations have been demonstrated in many chronic inflammatory states, such as obesity [25] and Alzheimer’s disease [26]. Monocytes have long been considered important to SCD pathophysiology; however, few studies have sought to characterize monocyte populations from SCD patients.

Most patients with SCD have monocytosis [7,27,28]. This finding is positively correlated with markers of hemolysis and negatively correlated with hemoglobin concentration, suggesting a worsening clinical course [29]. Similarly to SCD neutrophils, SCD monocytes are chronically activated, expressing a greater amount of CD11b on their surface and producing higher levels of interleukin (IL)-1b and tumor necrosis factor (TNF)-alpha than monocytes from healthy controls [30]. These cytokines activate the endothelium through the nuclear factor (NF)-kappa beta pathway, increasing endothelial expression of E-selectin, vascular adhesion molecule-1 (VCAM) and intracellular adhesion molecule-1 (ICAM) [30,31]. Importantly, sickle erythrocytes can form abnormal attachments with the endothelium through VCAM and ICAM [32]. These adhesion molecules also play key roles in leukocyte recruitment and attachment to the endothelium [33,34]. Thus, activated monocytes in SCD antagonize the endothelium and predispose its adherence to sickle RBC and activated leukocytes, heightening the risk for VOC.

SCD patients also suffer from intravascular hemolysis, which results in the release of hemoglobin and its breakdown product, heme, into the circulation. Excessive free heme causes oxidative damage and induces an inflammatory cascade that further irritates the endothelial lining [35], increases vascular remodeling [36], and induces vascular stasis [37]. Heme oxygenase-1 (HO-1) directs the body’s response to hemolysis by degrading heme into carbon dioxide, ferrous iron, and biliverdin. Induction of HO-1 has been shown to protect the endothelium against hemolysis and oxidative stress [38]. The human leukocyte with the highest HO-1 production is the circulating monocyte, specifically the nonclassical CD14dimCD16+ monocyte subset [39]. This subset, also known as endothelial patrolling monocytes (PMos), are intravascular housekeepers that surveil the endothelium for attached particles and phagocytose cellular debris from damaged vascular endothelium [40]. PMos in SCD patients express higher levels of HO-1 than in healthy individuals and SCD patients with recent VOC have depleted PMos levels [41]. Additionally, mice lacking PMos display more vascular stasis in the presence of sickle RBC than control mice [41]. Interestingly, the control phenotype is recoverable with reintroduction of PMos [41], suggesting that this subset plays a critical role in maintaining the integrity of SCD vasculature and preventing VOC. In this study, we sought to characterize the monocyte populations found in SCD patients by performing morphologic, enzymatic, and immunophenotypic analysis via flow cytometry on the peripheral blood of SCD patients hospitalized for VOC.

This article was previously presented as a poster at the American Society of Hematology Annual Meeting, December 2023.

## Materials and methods

Sample collection

Peripheral blood samples were obtained from 17 SCD patients during hospitalization for VOC. All patients were homozygotes for the sickle cell allele aside from one heterozygous patient with hemoglobin C disease. The patients included nine males and eight females ages 20-63. Peripheral blood was also obtained from 10 healthy volunteers ages 20-79. The white blood cell count showed a median value of 16,370 cells/mL in SCD patients and 9,250 cells/mL in controls. The absolute monocyte count ranged from 341 cells/mL to 2,576 cells/mL in SCD patients (median, 1105 cells/mL) and from 324 cells/mL to 1008 cells/mL in controls (median, 696 cells/mL). The samples were collected in ethylenediaminetetraacetate acid (EDTA) tubes and processed within six hours of venipuncture by the hematology laboratory of Case Western Reserve University at MetroHealth Medical Center Cleveland, Ohio. 

Specimen processing

Blood specimens were processed using a standard lysed whole-blood technique. 100 mL of blood was combined with 20 mL of each antibody except for My-4 for which only 5 mL was added. This mixture was then incubated first for 15 minutes at room temperature. A second incubation was performed in the dark after 2.0 mL of fluorescence-activated cell sorting (FACS) lysing solution (Becton Dickson Immunocytometry Systems, San Jose, CA, USA) was added to each tube and vortexed. The cells were then centrifuged for five minutes at 1200 rpm, washed in phosphate-buffered saline (pH 7.4) twice, and resuspended in .5 mL of .5% paraformaldehyde. 

All monoclonal antibodies were obtained from either Coulter Cytometry or Becton Dickinson Immunocytometry Systems. Automated cell blood counts (CBCs) (Sysmex XE 2100, Lincolnshire, IL, USA), mononuclear cell separation (Ficoll-Hypaque), and monoclonal antibody staining were completed within six hours. To obtain co-expression of monocyte antigens, each specimen was labeled with a three-color combination of the following monoclonal antiantibodies: (1) CD14 (My-4); (2) CD16 (Leu-11c); (3) CD4 (Leu-3a); or (4) anti-HLA-DR. Leu-11c and anti-HLA-DR were coupled with phycoerythrin (PE). Leu-3a was coupled with peridinin chlorophyll protein (PerCP). My-4 was coupled with fluorescein isothiocyanate (FITC). The fluorochrome compensation of each sample was adjusted with a combination of anti-CD8-FITC, anti-CD4-PerCP, and anti-CD19-PE. There were no electronic setting adjustments for the monocyte fraction analysis. 

Flow cytometry

Flow cytometry analysis was performed using a FACScan flow cytometer (Becton Dickinson) in the manufacturer-set configuration and a Consort 30 computer (HP 9000, model 310, Hewlett Packard Company, North Hollywood, CA, USA). Following electronic fluorochrome compensation adjustment, 10,000 events per sample were acquired. These events were analyzed using Paint-A-Gate and FACScan research software (Becton Dickinson). Five parameter histograms made up of six dot plots were used to display the fluorescence data in the Paint-A-Gait program. Weakly positive CD14-positive neutrophils were excluded using dual parameter gating. These gates were set by painting cells displaying light-scatter characteristics of monocytes with one color and CD14-positive cells with another color. Cells possessing both colors were selected for analysis using the Paint-A-Gait program and FACScan research software. Dual parameter histograms were created for PE vs FITC, PE vs PerCP, and FITC vs PerCP using FACScan research software. The percentage of positive cells for each set of monoclonal antibodies was determined by setting positive and negative quadrants and using appropriate fluorochrome-labeled isotype controls. 

Cytochemical staining

Buffy coat slides were prepared from 17 SCD patients and 10 healthy controls. Wright and α-naphthyl butyrate esterase (ANBE) stains were applied using standard procedure to both sets of slides to compare the morphological and cytochemical characteristics of SCD patients and control peripheral smears. The percentage of ANBE-positive monocytes was determined by counting 100 monocytes from each sample. 

Statistical methods

All quantitative data comparisons between patients and controls were made using an independent samples t-test. All analyses were performed using statistical software and statistical significance was evaluated at the 0.05 level. 

The protocol was submitted to the Institutional Review Board (IRB) at MetroHealth Medical Center in Cleveland, Ohio, where the study took place. After review, it was determined that the protocol qualified for exemption.

## Results

CBC findings during an acute VOC

**Figure 1:**
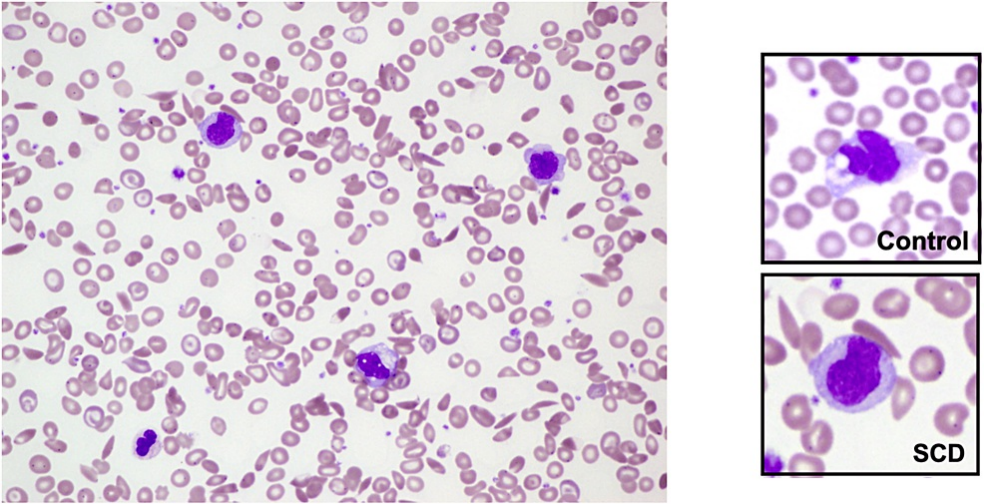
Wright stain (x60) showing leukocytosis and immature morphology of monocytes including high nuclear/cytoplasmic ratio, indented or less lobular nuclei and decreased cytoplasmic vacuolation. SCD = Sickle cell disease

SCD is known to be marked by absolute monocytosis and leukocytosis [7,28]. Here we demonstrate these findings in the setting of SCD both quantitatively (Table 1) and qualitatively (Figure 1). As expected, our patients had an increase in both monocytes and neutrophils, contributing to the greater levels of WBCs observed in SCD patients compared to healthy controls (Table 1). Our patients additionally had the anticipated decreases in RBC count and hemoglobin (Hb)/hematocrit (Hct) that are expected for SCD patients hospitalized for VOC.

**Table 1:** Comparison of automated complete blood count data between sickle cell disease patients and controls. RBC = Red Blood cells Hb = Hemoglobin Hct = hematocrit SCD = Sickle cell disease

	SCD	Control
WBC (/uL)	14,620*	8,370
Monocytes (/uL)	1,223*	671
Neutrophils (/uL)	9,348*	5,455
RBC (x10^6^/uL)	3.09*	4.26
Hb (g/dL)	8.04*	12.49
Hct (%)	23.43*	37.37
Platelet (/uL)	304,100	260,600

Immature morphology in SCD monocytes

The monocytes obtained from SCD patients’ peripheral blood were found to have features resembling monocytes in the blastic or promonocytic stages of development. These characteristics included high nuclear/cytoplasm ratio, indented or less lobular nuclei, and decreased cytoplasmic vacuolation (Figure 1). ANBE activity was also significantly decreased in SCD monocytes compared to control monocytes. 

Surface protein expression patterns from SCD patients and controls 

**Figure 2:**
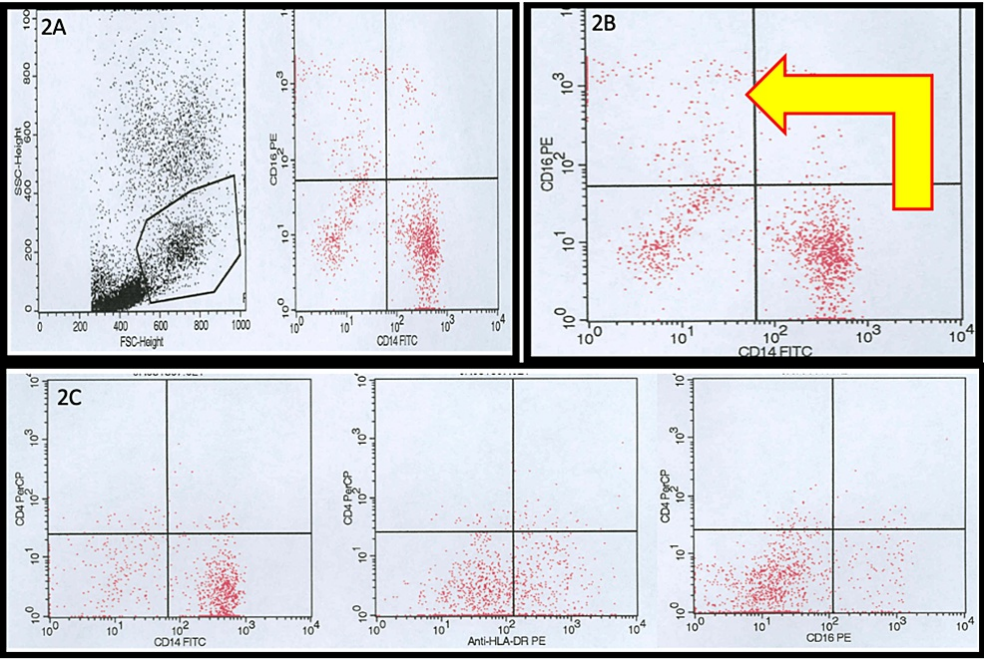
HLA-DR, CD4, CD14, and CD16 expression in 3-color flow cytometry from SCD patients (yellow arrow highlights CD14dimCD16+ monocyte subpopulation from SCD patients). PE= Phycoerythrin PerCP= Peridinin-Chlorophyll-Protein FITC = fluorescein isothiocyanate SSC = Side scatter FSC = Forward scatter HLA = Human leukocyte antigen SCD = Sickle cell disease

**Figure 3:**
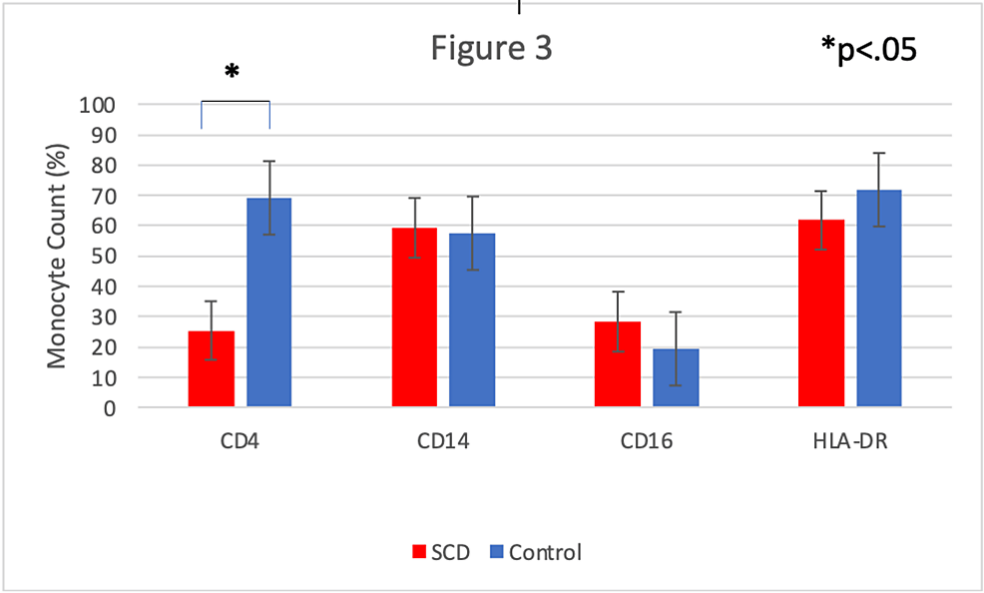
Comparison of HLA-DR, CD4, CD14 and CD16 expression from sickle cell disease patients and controls. CD = Cluster of differentiation HLA = Human leukocyte antigen SCD = Sickle cell disease

**Figure 4:**
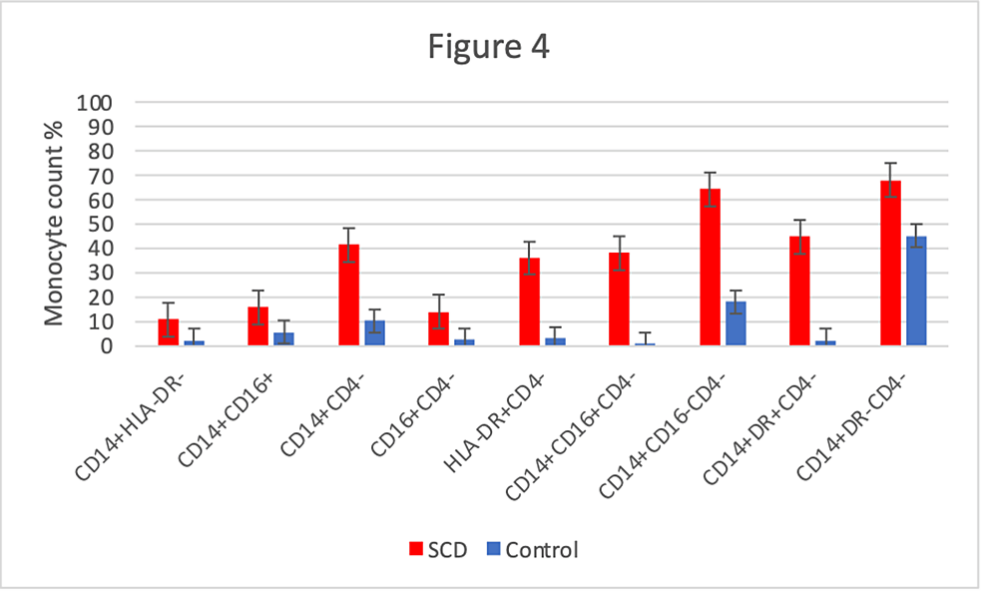
Comparison of expression patterns using 3-color flow cytometry from sickle cell disease patients and controls. All expression differences were found to be significant (p < .05) HLA = Human leukocyte antigen CD = Cluster of differentiation SCD = Sickle cell disease

Three-color flow cytometric analysis revealed a significant decrease in sickle monocyte co-expression of CD4 with CD14, HLA-DR, and CD16. This was observed both qualitatively (Figure 2C) and quantitatively (Figure 3). Predictably, we found concurrent increases in all CD4- monocyte subsets (Figure 4). This included significant increases in the CD14+CD16-CD4- (64.22% vs 18.09%) and CD14+CD4-HLA-DR- (67.91% vs 44.96%) monocyte fractions compared to controls (Figure 4). Finally, we also found a larger proportion of CD14+HLA-DR- (10.87% vs 2.19%) and CD14+CD16+ (15.88% vs 5.46%) SCD monocytes compared to healthy patients (Figure 4). Qualitatively, we can see the CD14+CD16+ subset is largely composed of CD14dimCD16+ monocytes (Figure 2).

## Discussion

The clinical course of SCD is punctuated by painful VOC. Recurrent VOC promotes pro-inflammatory changes in the immune profile that result in chronic activation of neutrophils and monocytes [6,7]. These innate immune cells actively contribute to microvasculature obstruction through their interactions with the vascular endothelium and sickle RBC [10,11]. Our study provides further evidence of monocyte abnormalities in SCD and sheds light on the monocytic contribution to VOC genesis. 

In our patient cohort, we observed an increase in WBCs that was largely due to increases in neutrophils and monocytes. These elevations along with the enhanced adhesive properties of these cells in SCD are well-described in the literature [10,11,30]. Moreover, high levels of neutrophils and monocytes are known to function as predictors of disease severity, indicating a worsening clinical course [13,29]. This could be partially explained by the reciprocal activating effects between monocytes and vascular endothelial cells. These effects likely generate a positive feedback loop wherein the endothelium recruits and stimulates monocytes from hematopoietic stores that then drive the chronic inflammation that characterizes SCD vasculature [30-32]. Interestingly, TNF-a blockers etanercept and infliximab have been shown to ameliorate monocyte activation and endothelial inflammation in sickle transgenic mice [42]. This study also found that the IL-1b receptor antagonist anakinra was less effective than TNF blockers in reducing monocyte activation and inflammation, suggesting that monocyte-derived TNF-a may be the sentinel cytokine in the SCD inflammatory axis [42].

The SCD monocytes we analyzed possessed an immature phenotype. This was demonstrated immunophenotypically by decreased CD4 expression. CD4 expression is known to be low in neonatal monocytes and is associated with a diminished capacity for antigen presentation [43,44]. Indeed, HLA class II molecules require CD4 costimulation during antigen presentation. Monocyte CD4 molecules are also thought to interact with the Fc receptors of other immune cells to facilitate clearance of pathogens and antigen-antibody complexes [45]. Monocytes from COVID-19 patients and post-trauma patients also have decreased CD4 expression, suggesting that the impaired immune response of SCD patients could be related to the immunological dysregulation observed in these disease states [46,47].

The immaturity of SCD monocytes was also demonstrated by decreased ANBE activity. ANBE is a plasma ectoenzyme thought to be involved in chemotaxis of mature monocytes [48]. Peripheral blood smear analysis revealed that SCD monocytes had less vacuoles and a smaller amount of cytoplasm when compared to healthy monocytes. These same enzymatic and morphologic findings are seen in the monocytes of newborn and post-trauma patients. Of note, both newborn and post-trauma patients are known to have slow, diminished immune responses and increased susceptibility to infection [49,50]. Previous studies have shown monocytes from post-trauma and newborn patients have diminished HLA-DR positivity. This made us curious about the HLA-DR expression levels of SCD monocytes. We found no significant difference in the HLA-DR expression pattern between SCD and control monocytes. However, we did observe significantly increased fractions of CD14+CD16-CD4- and CD14+CD4-HLA-DR- monocytes which are also seen in newborns and post-trauma patients [43,47]. To our knowledge, the morphologic, cytochemical and immunophenotypic properties of SCD monocytes during acute VOC have not been reported. 

Most abnormal monocyte subsets we observed in SCD patients can be explained by diminished CD4 positivity. However, the increases in CD14+HLA-DR- and CD14+CD16+ require additional consideration. CD14+HLA-DR- monocytes have exquisite immunosuppressive activity and have been found in increases proportions in the tumor microenvironment of pancreatic, prostate, and ovarian cancers [51-53]. Importantly, this subset is associated with disease progression, suggesting CD14+HLA-DR- monocytes could be partially responsible for the diminished immune surveillance that occurs in malignancy [52]. This subpopulation is also increased in viral infection and acute myocardial infarction which furthers the argument that these cells may trigger immune dysregulation in many disease states [54,55].

Recently, CD14dimCD16+ monocytes, also referred to as patrolling monocytes, have received a great deal of attention for their role in endothelial surveillance. These cells, specifically high HO-1 patrolling monocytes, have been shown to play an important role in protecting SCD vasculature and preventing VOC. While we did not measure HO-1 expression in this study, we can speculate that the CD14dimCD16+ subset we found likely possessed high HO-1 activity. Previous work has shown HO-1high patrolling monocytes are depleted following VOC. Herein, we demonstrate an elevation in patrolling monocytes during VOC which suggests these are likely the same cells being consumed during the VOC inflammatory cascade. 

Advancements in gene therapy and hematopoietic stem cell transplant are likely the future of curative SCD care. However, these therapies are not widely available due to an inadequate compatible donor population, cost, and other factors. Targeted immunotherapies against specific members of the innate immune system are a treatment modality showing promise in SCD. Currently, neutrophils and platelets are able to be pharmacologically restrained with pan-selectin inhibitors and ADP-receptor antagonists [27]. Crizanlizumab, a monoclonal antibody against p-selectin, has been shown to reduce the median rate of VOC by 45% compared to placebo [56]. Invariant natural killer T-cell depletion is another novel therapy being developed [27]. To our knowledge, there are not any therapies currently being trialed that specifically target monocytes. One therapy that may be useful in managing VOC is monocyte-specific leukocytapheresis. Indeed, extracorporeal elimination of pro-inflammatory monocytes is known to be efficacious in attenuating the immune response in refractory inflammatory conditions of the skin and gastrointestinal tract [57-59]. Previously, TNF-a producing CD14dimCD16+ monocyte apheresis has been performed in Ulcerative colitis patients [59]. Perhaps, a cytopheretic method could be added to the armamentarium of SCD therapies that removes CD11b+ SCD monocytes that produce TNF-a and/or IL-1b in patients who fail to respond to first- or second-line treatment.

## Conclusions

Taken together, our findings suggest that SCD patients mobilize relatively immature monocytes that possess a lower capacity for antigen presentation with the potential for immune dysfunction. However, the conclusions from our work are weakened by a relatively small sample size and lack of correlation to long-term outcomes. More investigation is needed to elucidate the precise molecular mechanisms of monocyte involvement in preventing, generating, and resolving VOC. While we wait for widespread curative SCD treatments, better anteroom therapies are needed. Monocyte-specific leukocytopheresis may be one such treatment that could meaningfully diminish the morbidity and mortality of SCD in patients without access to gene therapy or transplant. 
